# Exploration of CT Images Based on the BN-U-net-W Network Segmentation Algorithm in Glioma Surgery

**DOI:** 10.1155/2022/4476412

**Published:** 2022-04-11

**Authors:** Yongmei Yu, Zhaofeng Du, Changxin Yuan, Jian Li

**Affiliations:** ^1^Department of Radiology, Jining First People's Hospital, No. 6 Jiankang Road, Jining, Shandong 272000, China; ^2^Department of Imaging, Zaozhuang Traditional Chinese Medicine Hospital, No. 2666 Taihangshan South Road,Xuecheng District, Zaozhuang 277000, Shandong, China; ^3^Military Sursery, Zaozhuang Traditional Chinese Medicine Hospital, No. 2666 Taihangshan South Road,Xuecheng District, Zaozhuang, Shandong, China

## Abstract

This study aimed to explore the application value of computed tomography (CT) imaging features based on the deep learning batch normalization (batch normalization, BN) U-net-W network image segmentation algorithm in evaluating and diagnosing glioma surgery. 72 patients with glioma who were admitted to hospital were selected as the research subjects. They were divided into a low-grade group (grades I-II, *N* = 27 cases) and high-grade group (grades III-IV, *N* = 45 cases) according to postoperative pathological examination results. The CT perfusion imaging (CTPI) images of patients were processed by using the deep learning-based BN-U-net-W network image segmentation algorithm. The application value of the algorithm was comprehensively evaluated by comparing the average Dice coefficient, average recall rate, and average precision of the BN-U-net-W network image segmentation algorithm with the U-net and BN-U-net network algorithms. The results showed that the Dice coefficient, recall, and precision of the BN-U-net-W network were 86.31%, 88.43%, and 87.63% respectively, which were higher than those of the U-net and BN-U-net networks, and the differences were statistically significant (*P* < 0.05). Cerebral blood flow (CBF), cerebral blood volume (CBV), and capillary permeability (PMB) in the glioma area were 56.85 mL/(min·100 g), 18.03 mL/(min·100 g), and 8.57 mL/100 g, respectively, which were significantly higher than those of normal brain tissue, showing statistically significant differences (*P* < 0.05). The mean transit time (MTT) difference between the two was not statistically significant (*P* > 0.05). The receiver operating characteristic (ROC) curves of CBF, CBV, and PMB in CTPI parameters of glioma had area under the curve (AUC) of 0.685, 0.724, and 0.921, respectively. PMB parameters were significantly higher than those of CBF and CVB, and the differences were statistically obvious (*P* < 0.05). It showed that the BN-U-net-W network model had a better image segmentation effect, and CBF, CBV, and PMB showed better sensitivity in diagnosing glioma tissue and normal brain tissue and high-grade and low-grade gliomas, among which PBM showed the highest predictability.

## 1. Introduction

Glioma is a common malignant tumor in the cranial nervous system, and its incidence accounts for about 40% of all intracranial tumors. At present, the pathogenesis of the disease is not clear. The common types are astrocytoma, oligodendroglioma, ependymoma, neuronal tumor, and medulloblastoma [1, 2]. Due to the different locations of the tumor in the brain, the clinical symptoms are also different. Most patients have headache, vomiting, and memory loss as the main symptoms of increased intracranial pressure [3]. Most of glioma grow infiltratingly, so the boundary with normal brain tissue is unclear; and the higher the tumor grade, the more unclear the boundary, the more serious the infiltration of peritumoral brain tissue [4, 5]. According to the classification criteria of glioma issued by the World Health Organization (WHO), it can be divided into grades I–IV, which were benign, borderline with a trend towards III and IV, and malignant [6]. Surgical resection is the main method for the treatment of glioma, but due to the different tumor grades, the prognosis varies greatly. Therefore, obtaining accurate glioma classification information is of great significance for the selection of treatment methods, surgical guidance, and the improvement of prognosis [6–8].

Clinically, the commonly used diagnostic methods for glioma are computed tomography (CT), magnetic resonance imaging (MRI), and other imaging examinations. However, the imaging characteristics of glioma lack specificity. The imaging of grades I–IV can be manifested as mixed signal images with different degrees of necrosis or cystic transformation. Therefore, conventional imaging examinations cannot accurately provide specific conditions that are not conducive to glioma grading, such as tumor angiogenesis, metabolism, and micronecrosis [9–12]. In recent years, CT perfusion imaging (CTPI) has shown superiority in reflecting the blood perfusion of tumors. Clinical scholars have used it in the diagnosis and classification of glioma patients, and the results have been significantly improved [13, 14]. However, each patient's craniocerebral condition is complex and changeable, with different manifestations in CT imaging. Clinicians diagnose and analyze the patient's condition by observing and analyzing each CT image. They are easily affected by certain objective factors, such as the doctor's experience, mental state, and the quality of CT imaging. This is not only time-consuming and labor-intensive but also causes risk of misdiagnosis and missed diagnosis [15–17].

With the development of science and technology and the enhancement of artificial intelligence computing capabilities, deep learning has begun to be widely used in visual image processing, data mining, and other fields and has achieved good results [18]. In clinical practice, some scholars use deep learning technology to analyze medical image maps to assist doctors in segmenting lesions, completing target detection and classification, and achieving good results [19, 20]. However, the traditional deep learning technology is difficult to achieve the purpose of more complex image segmentation such as glioma. Therefore, a 128-slice CT whole brain perfusion image segmentation method based on the U-net network was proposed in this study, which was used in the diagnosis and analysis of glioma patients.

In conclusion, obtaining accurate glioma grading information is of great significance for the selection of treatment methods, the guidance of surgery, and the improvement of prognosis. However, the U-net network in the clinical deep learning technology has shown good results in the processing of medical CT images at present. Therefore, this work proposed a segmentation algorithm of CT perfusion images based on the U-net network and applied it to the clinical diagnosis and analysis of glioma patients. It was hoped to explore the clinical practical value of the algorithm by comparing the imaging effects with the U-net and BN-U-net network algorithms that have been clinically proven to have good image segmentation effects, providing an effective reference for the diagnostic analysis and surgical treatment of glioma patients.

## 2. Materials and Methods

### 2.1. Research Objects and Their Grouping

In this study, 72 patients with glioma admitted to the hospital from July 2018 to June 2020 were selected as the research subjects. All patients were confirmed to be glioma by postoperative pathological biopsy and immunohistochemistry. Among them, 40 were males and 32 were females; and patients were 15–76 years old (with an average value of 42.75 ± 14.32 years old). All patients underwent 128-slice CT whole brain perfusion scan (Siemens SOMATOM definition AS 128-slice spiral CT machine) before surgery, and pathological examination was performed after operation to determine the pathological type and pathological grade. 72 patients were rolled into two groups based on postoperative pathological examination results and WHO pathological grading standards: 27 cases in the low-grade group (I-II) and 45 cases in the high-grade group (III-IV). This study had been approved by the ethics committee of hospital, and the patients and their families had understood the situation of the study and signed the informed consent forms.

Inclusion criteria: patients who were diagnosed as primary glioma by pathological examination, patients whose imaging data were well preserved, and patients with no contraindication to CT examination.

Exclusion criteria: patients who were allergy to iodine contrast agent, patients with hyperthyroidism, patients with intracranial tumor metastasis or multiple intracranial tumors, patients in the late stage of glioma with obviously increased intracranial pressure, patients with tendency to brain herniation, and patients with cardiopulmonary insufficiency.

### 2.2. CTPI

Before scanning, the patients had an iodine allergy test. Allergy rescue materials were prepared (epinephrine, dexamethasone, and nasal oxygen tube) for rescue at any time. 0.1 mL of iodine contrast agent was adopted for intradermal injection, and whether the subjects had allergic reactions should be observed after 15–20 minutes. The elbow vein was punctured with a 16G needle to ensure that the contrast agent was injected quickly and stably. The patient was instructed to lie supine, a routine brain scan was performed with the ear canthus line as the baseline to determine the extent of the lesion, and then a CT whole brain perfusion scan was performed. A high-pressure syringe was used to inject the contrast medium through the cubital vein at a speed of about 5.5 mL/s according to the subject's weight (1–1.5 mL/kg). Then, centering on the lesion, a continuous dynamic scan of 96 mm of the whole brain was performed. The scanning parameters were set as follows: scanning time was 40 seconds, scanning layer thickness was 0.6 mm, layer thickness was 1.0 mm, tube voltage was 80 kV, tube current was 120 mA, and 0.33 seconds for 1 cycle of tube rotation.

A three-dimensional postprocessing workstation was adopted to process the acquired data with a whole brain volume perfusion software package to obtain the pseudocolor maps of cerebral blood flow (CBF), cerebral blood volume (CBV), mean transit time (MTT), and capillary permeability (PMB).

### 2.3. Glioma CT Image Segmentation Algorithm Based on the U-net Network

The U-net network is an extension of the full convolutional neural network (FCN), which can be divided into two parts. The first half was used to extract image features, and the second half was used for sampling. Taking into account that the U-net network may have slower convergence speed and disappearance of gradients during training, the research had added a part of the norm layer to normalize it. At the same time, the research replaced the ordinary convolution in the U-net network with the depth separable convolution to improve the calculation speed, which improved the calculation speed of the model by reducing the network parameters and reducing the size of the network model. Therefore, a BN-U-net-W network model was designed.

The batch normalization (BN) was adopted for the normalization process to process the input value distribution of any neuron in each layer of the neural network into a standard normal distribution with a mean of 0 and a variance of 1, so as to improve the convergence speed and shorten the training time of the model. The process of BN normalization processing was mainly as follows.

It was assumed that the input was *s*, which was the number of all samples, and *β* was the parameter. If *α*={*x*_1_ … *x*_*s*_}, the equation for calculating the mean value of the batch data can be expressed as follows:

(1) $$\begin{array}{c} \begin{array}{c} \rho_{\alpha }=\frac{1}{s}\sum_{i=1}^{s}x_{1} \end{array}. \end{array}$$

The data variance of each training batch can be expressed as

(2) $$\begin{array}{c} \sigma_{\alpha }^{2}=\frac{1}{s}\sum_{i=1}^{s}{\left(x_{i}-\rho_{\alpha }\right)}^{2}. \end{array}$$

The mean and variance of the data were adopted to perform BN on the training data to obtain a 0-1 normal distribution. In addition, *ε* was adopted to represent a very small positive number; then, the equation can be expressed as

(3) $$\begin{array}{c} \hat{x}=\frac{x_{i}-\rho_{\alpha }}{\sqrt{\sigma_{\alpha }^{2}+\varepsilon }}. \end{array}$$

It could multiply *x*_*i*_ by *γ* to adjust the size of the integer value and then add *α* to increase the offset to get *m*_*i*_, where *γ* represents the scale factor, *α* represents the translation factor, and *m*_*i*_ can be expressed as

(4) $$\begin{array}{c} m_{i}=\gamma {\hat{x}}_{i}+\alpha . \end{array}$$

**Figure 1:**
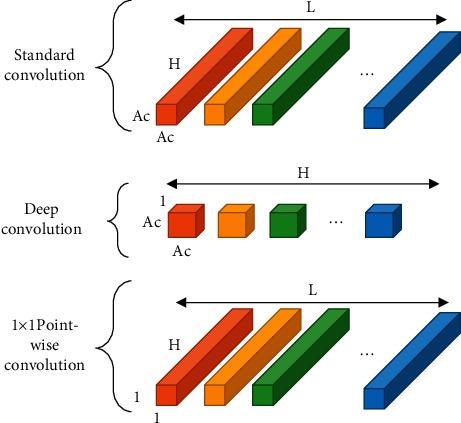
Schematic diagram of depth separable convolution.

Depth separable convolution can decompose the standard convolution into depth convolution and an *l* × 1 point-by-point convolution. The principle is shown in Figure 1. Let *A*_*c*_ be the spatial dimension of the convolution kernel, and *P*_*v*_ be the size of feature map inputted by the convolution layer. The calculation amount of the standard convolution is as follows:

(5) $$\begin{array}{c} \text{Standard convolution}=A_{c}\times A_{c}\times H\times L\times P_{v}\times P_{v}. \end{array}$$

The amount of calculation for deep convolution is as follows:

(6) $$\begin{array}{c} \text{Deep convolution}=A_{c}\times A_{c}\times P_{v}\times P_{v}. \end{array}$$

The calculation amount of 1 × 1 point-by-point convolution is as follows:

(7) $$\begin{array}{c} 1\times 1\text{ pointwise convolution}=H\times L\times P_{v}\times P_{v}. \end{array}$$

Based on equations (5)–(7), the calculated ratio of depth separable convolution nuclear standard volume data can be obtained:

(8) $$\begin{array}{c} \frac{1}{L}+\frac{1}{A_{c}^{2}}=\frac{A_{c}\times A_{c}\times H\times P_{v}\times P_{v}+H\times L\times P_{v}\times P_{v}}{A_{c}\times A_{c}\times H\times L\times P_{v}\times P_{v}}. \end{array}$$

**Figure 2:**
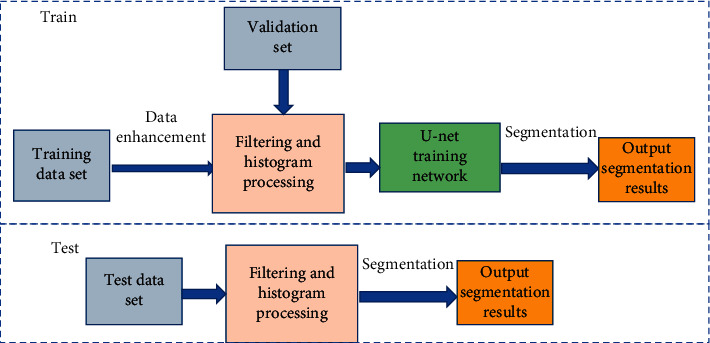
The training process of the BN-U-net-W network.

The training dataset of the BN-U-net network model was the CT image, and the glioma standard was performed by experienced clinicians. Network training used NVIDIA GTX Titan V GPU for acceleration, the number of iterations was set to 50 epochs, the learning rate was 0.001, the batch size was set to 4, and the optimization function was RMSP_rop_. The training process is shown in Figure 2. The training dataset was input to the BN-U-net network model for training after data enhancement, filtering, and histogram, and the segmentation results were output after segmentation. After the test dataset was filtered and histogram processed, it was directly segmented to output the segmentation results.

### 2.4. Observation Indicators

To compare the sensitivity, specificity, and accuracy of CTPI to the pathological classification of glioma, TP was used as the true positive of the test result, FN was false negative, FP was false positive, and TN was true negative:

(9) $$\begin{array}{c} \text{Accuracy}=\frac{TP+TN}{TP+TN+FP+FN},\\ \text{Sensitivity}=\frac{TP}{TP+FN},\\ \text{Specificity}=\frac{TN}{FP+TN}. \end{array}$$

The effect of CTPI image segmentation was evaluated mainly using Dice similarity coefficient (DSC), recall, and precision, which could be calculated as follows:

(10) $$\begin{array}{c} DSC=\frac{2\left|Z\cap D\right|}{\left|Z\right|+\left|D\right|}. \end{array}$$

In equation (10), *Z* was the result of manual segmentation by experts, and *D* was the segmentation result of the algorithm designed in this research. The value of Dice coefficient ranged from 0 to 1. The larger the value, the better the segmentation effect of the algorithm. The smaller the value, the poor the performance of the algorithm's glioma segmentation or the segmentation error. Precision and recall are calculated as follows.

(11) $$\begin{array}{c} \begin{array}{c} \text{Precision}=\frac{TP}{TP+FP}, \end{array}\\ \text{Recall}=\frac{TP}{TP+FN}. \end{array}$$

### 2.5. Statistical Analysis

SPSS 18.0 statistical software was adopted for data analysis, and the measurement data were expressed as mean ± standard deviation ($\bar{x}\;$ ± *s*). The independent sample *t*-test was used for comparison between groups, and *P* < 0.05 was considered statistically significant. The receiver operating characteristic (ROC) analysis was used to calculate the area under the ROC curve (AUC) and compare the diagnostic accuracy of CTPI parameters. The maximum value of Youden index (sensitivity + specificity-1) was selected as the cutoff point for diagnosing high and low-grade glioma, and the corresponding sensitivity and specificity were calculated.

## 3. Results

### 3.1. Basic Data

**Figure 3:**
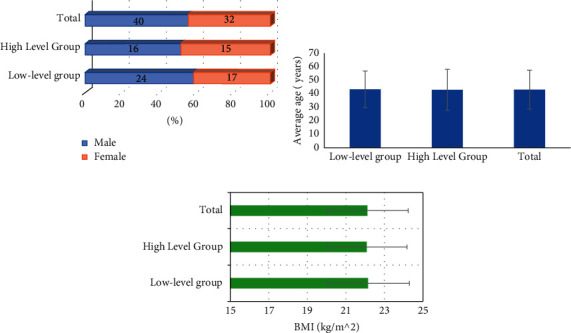
Comparison of the general data of the two groups of patients. (a) The gender distribution of the two groups of patients. (b) The comparison of the average age of the two groups of patients. (c) The comparison of the BMI of the two groups of patients.

Figure 3 shows a comparison chart of the general data of the two groups of patients. As illustrated in the figure, the number of male patients in the low-dose group and the high-dose group was 24 and 16, respectively, and the number of female patients was 17 and 15, respectively. The average ages of the patients in the two groups were 42.96 ± 13.45 years old and 42.57 ± 15.13 years old, respectively; and the body mass index (BMI) was 22.15 ± 2.14 kg/m^2^ and 22.08 ± 2.09 kg/m^2^, respectively. After comparison, it was found that there was no significant difference in the ratio of male to female, average age, and BIM between patients in the two groups (*P* > 0.05).

### 3.2. The Processing Results of the BN-U-net-W Network Model

**Figure 4:**
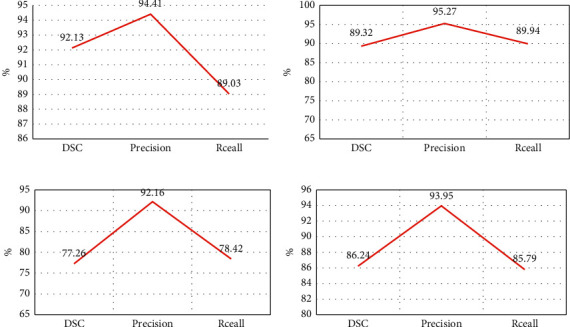
Processing results of the BN-U-net-W network model. (a)–(c) The performance analysis result of training set 1, training set 2, and training set 3, respectively. (d) The average result of performance analysis of three training sets.

The Dice coefficient, recall rate, and precision of the BN-U-net-W network model in training sets 1, 2, and 3 were compared, and the results are shown in Figure 4. As shown in Figure 4, the Dice coefficient, recall rate, and precision of the BN-U-net-W network model algorithm in training set 1 were 92.13%, 94.41%, and 89.03%, respectively. In training set 2, the Dice coefficient, recall rate, and precision were 89.32%, 95.27%, and 89.94%, respectively. In training set 3, the Dice coefficient, recall rate, and precision were 77.26%, 92.16%, and 78.42%, respectively. Figure 4(d) shows the average values of Dice coefficient, recall rate, and precision for the three training sets, which were 86.24%, 93.95%, and 85.79%, respectively.

### 3.3. Comparison on Performances of Different Algorithms

**Figure 5:**
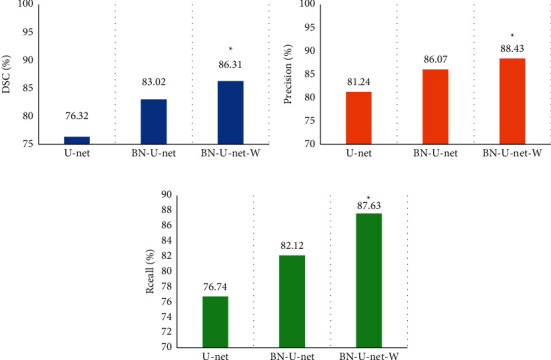
Comparison on performances of different algorithms. (a)–(c) The comparisons of Dice coefficient, recall, and precision, respectively. ^∗^The difference was statistically obvious in contrast to the BN-U-net-W algorithm (*P* < 0.05).

In order to verify the performance of the algorithm, the U-net [21] and BN-U-net [22] networks were introduced and compared with the proposed algorithm in terms of the average Dice coefficient, average recall, and average precision of the three when testing 400 glioma CT images. The results are shown in Figure 5. The Dice coefficient, recall, and precision of the BN-U-net-W network were 86.31%, 88.43%, and 87.63%, respectively, which were higher than those of U-net and BN-U-net networks, and the differences were statistically significant (*P* < 0.05).

**Figure 6:**
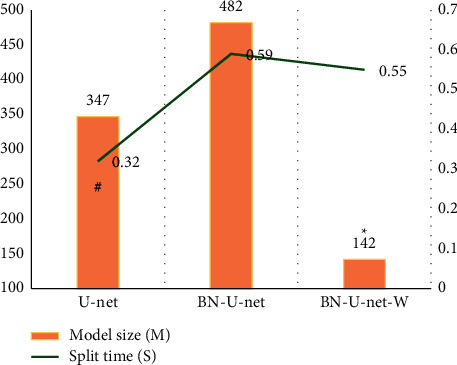
The model sizes and the shortest time of the three networks of three algorithms. ^∗^ and # indicate that the difference was statistically great compared with BN-U-net-W and U-net, respectively (*P* < 0.06).

The model sizes and the shortest time of the three networks required to segment a glioma CT image were calculated. The results are shown in Figure 6. Among the three types of networks, the U-net network took the shortest time to split a glioma CT, followed by the BN-U-net-W network, and the BN-U-net network took the longest time to be 0.59 seconds. Among the three networks, the model of the BN-U-net-W network was 142 M, which was significantly smaller than the other two, and the difference was statistically significant (*P* < 0.05).

### 3.4. Comparison of CTPI Parameters between Normal Brain Tissue and Glioma Area

**Figure 7:**
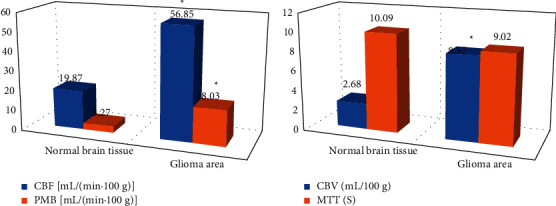
Comparison on CTPI parameters between normal brain tissue and glioma area. (a) The comparisons of CBF and PMB. (b) The comparisons of CVB and MTT. *∗*The difference was greatly significant compared with the glioma area (*P* < 0.05).

It statistically analyzed the CBF, PMB, CVB, and MTT of normal brain tissue and glioma tissue of 72 glioma patients, and the results are shown in Figure 7. The CBF, PMB, and CVB of glioma tissue were 56.85 (mL/(min 100 g)), 18.03 (mL/(min 100 g)), and 8.57 (mL/100 g), respectively, which were significantly higher than those in the normal brain tissue (19.87 (mL/(min·100 g)), 3.27 (mL/(min·100 g)), and 2.68 (mL/100 g), respectively), and the difference was statistically significant (*P* < 0.05). The MTT of normal brain tissue and glioma region of patients was 10.09 s and 9.02 s, respectively, and there was no significant difference between the two (*P* > 0.05).

### 3.5. Comparison on CTPI Parameters of Low-Grade and High-Grade Normal Brain Tissues

**Figure 8:**
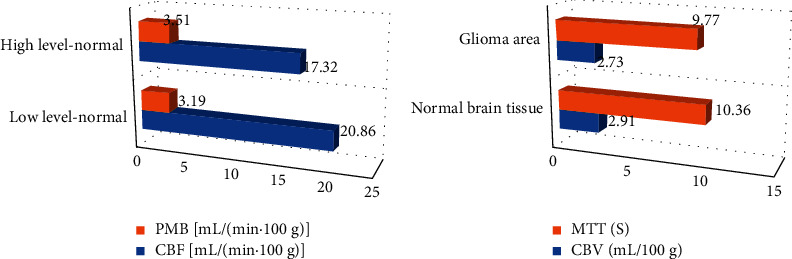
Comparison on CTPI parameters of low-grade and high-grade normal brain tissues. (a) The comparisons of CBF and PMB. (b) The comparisons of CVB and MTT.

A total of 27 cases in the low-grade group (grades I-II) and 45 cases in the high-grade group (III-IV) of CBF, PMB, CVB, and MTT were counted in the study. The results are shown in Figure 8. There was no obvious difference in CBF, PMB, CVB, and MTT between low-grade normal brain tissue and high-grade normal brain tissue, and there was no statistical significance (*P* > 0.05).

### 3.6. Comparison on CTPI Parameters between Low-Grade and High-Grade Glioma Areas

**Figure 9:**
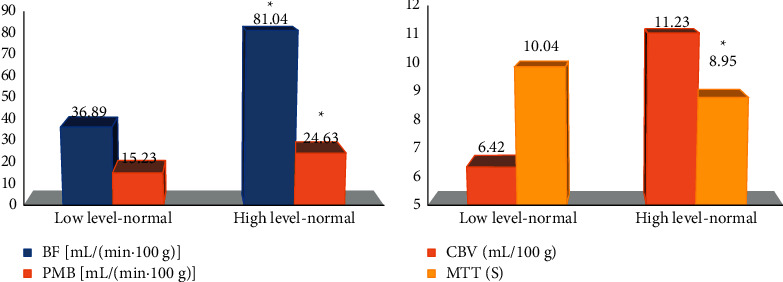
Comparison on CTPI parameters between low-grade and high-grade glioma areas. (a) The comparisons of CBF and PMB. (b) Comparisons of CVB and MTT. *∗*The difference was greatly significant compared with the glioma area (*P* < 0.05).

A total of 45 cases in the low-grade group (grades I-II) and 45 cases in the high-grade group (III-IV) of CBF, PMB, CVB, and MTT were counted in the study. The results are shown in Figure 9. The CBF, PMB, and CVB in the glioma area were 81.04 mL/(min·100 g), 24.63 mL/(min·100 g), and 11.23 mL/100 g, respectively, which were significantly higher than those of normal brain tissue, and they were statistically significant (*P* < 0.05). There was no significant difference in MTT between the two (*P* > 0.05).

### 3.7. ROC Analysis on CTPI Parameters between Low-Grade and High-Grade Glioma Areas

**Figure 10:**
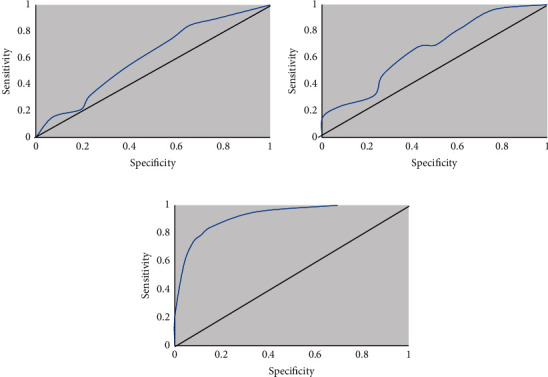
ROC analysis on CTPI parameters between low-grade and high-grade glioma areas. (a)–(c) The ROC results of CBF, CBV, and PMB, respectively.

The study analyzed the ROC curves of CBF, CBV, and PMB in the high-grade and low-grade glioma CTPI parameters, and the results are shown in Figure 10. The AUC of CBF, CBV, and PMB was 0.685, 0.724, and 0.921, respectively.

### 3.8. Imaging Data of Patients

**Figure 11:**
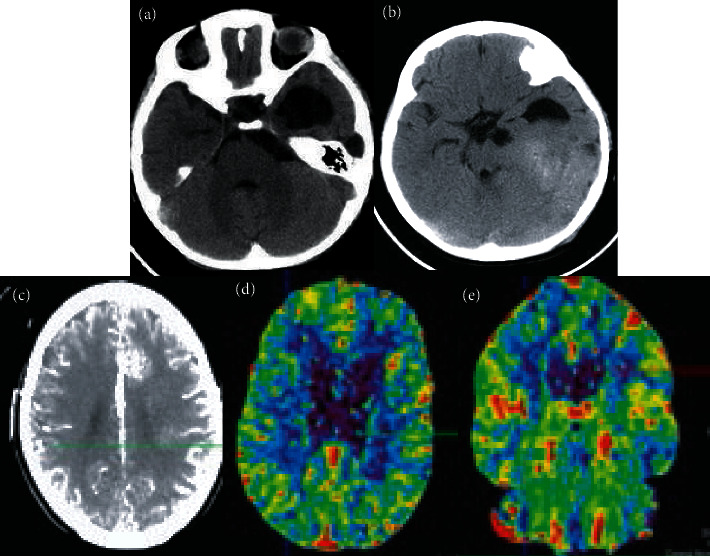
Imaging data of patients. (a)-(b) Ordinary CT. (c) Cranial enhanced CT. (d)-(e) CTPI.

Figure 11 shows the CT imaging data of a 68-year-old female patient. The clinical symptoms of the patient were headache and nausea for 2 weeks, which worsened for two days. Figures 11(a) and 11(b) show the normal CT, Figure 11(c) shows a brain enhanced CT, and Figures 11(d) and 11(e) show CT perfusion imaging. The figures demonstrated that the patient's glioma showed mixed density shadows, the glioma was irregular in shape, the boundary was unclear, and there were a small amount of cystic degeneration, hemorrhage, and necrosis. On contrast-enhanced CT, there were uneven enhancement, cystic degeneration, and hemorrhage, which showed that the patient's cerebral blood flow and surface infiltration were significantly aggravated.

## 4. Discussion

Glioma is a relatively common type of brain malignant tumors, which mainly occur in the central nervous system. Investigations have shown that the disease accounts for 40–50% of primary central nervous system tumors [7, 8]. The pathogenesis of glioma is not clear, but it is highly aggressive and has a high recurrence rate, so the clinical mortality rate is relatively high. Among the common glioma cell types, glioblastoma is the most common, accounting for about 50% of all gliomas, and the prognosis is extremely poor, with a recurrence rate close to 100% [23]. How to accurately obtain the specific type and classification of the patient's glioma is of great significance. Therefore, this study proposed a 128-slice CT whole brain perfusion image segmentation method based on the U-net network. In order to avoid the slowing down of the algorithm and the disappearance of the gradient, the research had added part of the norm layer and normalized it. At the same time, the ordinary convolution in the U-net network was replaced with depth separable convolution, which improved the speed of calculation, so a BN-U-net-W network model was designed.

In this study, the Dice coefficient, recall, and precision of the BN-U-net-W network model were calculated, and the average values were 86.24%, 93.95%, and 85.79%, respectively. In addition, in order to verify the performance of the algorithm, the study also introduced U-net and BN-U-net networks and compared the average Dice coefficient, average recall, and average precision of 400 glioma CT images tested by three network models. The results showed that the Dice coefficient, recall, and precision of the BN-U-net-W network were 86.31%, 88.43%, and 87.63%, respectively, which were higher than those of the U-net and BN-U-net networks, showing statistically obvious differences (*P* < 0.05). Such results were similar to the results of Wang et al. (2020) [24]. The BN-U-net-W network is added a norm layer to the full convolutional neural network (FCN) for normalization, which solved the problem that the U-net network may experience a decrease in convergence speed during training and testing. Slowness and gradient disappearance, in addition, the use of depthwise separable convolutional networks to replace ordinary convolutions can greatly reduce the size of the network model, reduce the parameters used in the network operation process, and greatly shorten the running time.

In addition, it also counted the model sizes of the three networks and the shortest time required to segment a glioma CT image in this study. Among them, the U-net network required the shortest time to segment a glioma CT, followed by the BN-U-net-W network. The longest time required for the BN-U-net network was 0.59 seconds; the model of the BN-U-net-W network was 142 M, which was much smaller than the other two, and the difference was statistically significant (*P* < 0.05). The study statistically analyzed the CTPI parameters of normal brain tissue and glioma area of 72 glioma patients. The results showed that the CBF, PMB, and CVB of glioma area were 56.85 mL/(min·100 g), 18.03 mL/(min·100 g), and 8.57 mL/100 g, respectively, which were much higher than those of normal brain tissue, and the differences were statistically significant (*P* < 0.05). This shows that these three parameters have better sensitivity in the diagnosis of glioma tissue and normal brain tissue, high-grade, and low-grade gliomas. The results of the study by Ahmad et al. (2016) [25] are also consistent with this work, indicating that CBF, PMB, and CVB show good detection sensitivity as monitoring indicators of neovascularization in patients with clinical glioma. In addition, using the correlation between the above indicators and glioma, the biological behavior of patients can be well evaluated. Finally, the study analyzed the ROC curves of CBF, CBV, and PMB in the high-grade and low-grade glioma CTPI parameters, and the AUCs were 0.685, 0.724, and 0.921, respectively. PMB parameters were significantly higher than CBF and CVB, and the differences were statistically significant, which showed that PBM had high predictability.

## 5. Conclusion

This study proposes a CT perfusion image segmentation algorithm based on the BN-U-net network and applies it to evaluate the application value of CT images in the diagnosis of glioma diseases. In order to verify the performance of the algorithm, the U-net and BN-U-net networks were introduced and compared with other algorithms in terms of Dice coefficient, recall, and precision. The results showed that the image segmentation effect of the BN-U-net-W network model designed in this study was better. Among the glioma CTPI parameters, CBF, CBV, and PMB were more sensitive in diagnosing glioma tissue and normal brain tissue and high-grade and low-grade glioma, and PBM had high predictability. However, this study did not make a detailed division of the pathological types of the study subjects and ignored the influence of different pathological types on CTPI parameters. In the follow-up study, the sample size can be increased to explore the impacts of different pathological types on CTPI parameters. In general, this study provided an effective reference for improving the survival rate and quality of life of patients with glioma.

## Data Availability

The data used to support the findings of this study are available from the corresponding author upon request.
